# Gender Dysphoria and Dissociative Identity Disorder: A Case Report and Review of Literature

**DOI:** 10.1016/j.esxm.2022.100553

**Published:** 2022-08-20

**Authors:** Lorenzo Soldati, Roland Hasler, Nathalie Recordon, Marie Clement, John Köhl, Nader Perroud

**Affiliations:** 1 Sexual medicine and sexology unit, Division of Psychiatric Specialties, Department of Psychiatry, University Hospital of Geneva, Geneva, Switzerland; 2 Department of Psychiatry, Faculty of Medicine, University of Geneva, Geneva, Switzerland; 3 TRE Unit, Division of Psychiatric Specialties, Department of Psychiatry, University Hospital of Geneva, Geneva, Switzerland; 4 Department of Psychiatry, Dalhousie University, Halifax, Scotia, Canada

**Keywords:** Gender Dysphoria, Dissociative Identity Disorder, Mental Health Assessment, Mental Health Care, Systematic Review

## Abstract

**Background:**

World Professional Association for Transgender Health guidelines support the importance of a mental health assessment before providing medical treatment for Gender Dysphoria (GD). During this assessment, patients without GD but with mental health disorder and who request treatment for GD should be excluded. Dissociative Identity Disorder (DID) is a poorly known mental disorder which can be confused for GD.

**Aim:**

To provide a case report of a patient suffering for DID but asking for treatment for GD and to provide a review of the literature on GD and DID.

**Method:**

A case report assessment and follow-up was described and a systematic review of the literature was performed in Pubmed, PsychInfo, and Embase databases.

**Outcome:**

To provide all cases with assessment and follow-up of DID and GD.

**Results:**

The case report describes a man suffering from DID and asking for hormonal treatment for GD. After assessment the patient was able to let go of his wish for treatment for GD and begin psychotherapy for DID. During the literature review 11 articles were included. 3 articles showed a prevalence of DID of 0%, 0.8% and 1,5% in GD samples. 5 articles were case reports of patients with both diagnoses of GD and DID which showed the complexity of the care of these patients. 2 articles were case reports, where a GD diagnosis was done first, and then counseling for GD was proposed. After a second session, the diagnosis was changed for DID. In 1 other case report and our case report there was a description of 2 persons suffering from DID and asking for treatment for GD.

**Clinical implications:**

Our review shows the complexity of providing care to patients with a comorbidity of GD and DID, as well as the complexity of making the differential diagnosis between GD and DID.

**Strengths and Limitations:**

A systematic review was performed on these rare cases. Our study presents the results for a small group of patients.

**Conclusions:**

This article provides the first systematic review on GD and DID and shows that DID in a GD sample does not seem to be higher than in the general population. In addition, it allow clinicians to gain better knowledge about patients suffering from both DID and GD and patients suffering from DID who ask for GD treatment.

**Soldati L, Hasler R, Recordon N, et al. Gender Dysphoria and Dissociative Identity Disorder: A Case Report and Review of Literature. Sex Med 2022;10:100553.**

## Introduction

The DSM 5^1^ defines Gender Dysphoria (GD) as a marked incongruence between one's experienced/expressed gender and one's assigned gender and/or one's primary and/or secondary sex characteristics. Individuals suffering from GD may express a strong desire to be rid of their primary and/or secondary sex characteristics and a strong desire for primary and/or secondary sex characteristics more congruent with their gender.

In the 11th edition of ICD, GD is classified in the category “Conditions related to sexual health” and renamed «Gender incongruence (GI)».^2^

Both assigned male at birth (AMAB) and assigned female at birth (AFAB) individuals suffering from GD may benefit from different treatments. There are hormonal therapies (HT) and gender affirming surgeries (GAS).^3^

Professionals involved in the care of persons with GD largely follow the standards of care developed by the World Professional Association for Transgender Health (WPATH) which are based on a somatic and a mental health assessment (MHA) before the initiation of a medical treatment for GD.^3^ The role of the MHA is to confirm that the patient fulfills DSM or ICD criteria for GD or GI and to exclude that the gender incongruence is not accounted for by a mental disorder or other conditions.^3^ Specialists suggest that the differential diagnosis must investigate conditions such as simple nonconformity to gender roles, body dysmorphic disorder, transvestic disorder or gender-themed delusions occurring in psychotic disorders.^4^ Moreover, the assessment of patients seeking treatment for HT and/or GAS but who do not clearly meet criteria for GD, may require more time.^4^ The same is true for those for whom the onset of GD is in the context of a mental disorder. A coexisting mental disorder is not a contraindication for HT or GAS if it is sufficiently controlled and does not interfere with the patient's capacity for decision-making or ability to safely adhere to the demands of the desired treatment.^4^

It is not specifically mentioned in the current guidelines which stress the importance of MHA in GD treatment^3^, ^4^, ^5^, ^6^ that Dissociative Identity Disorder (DID) is a possible differential diagnosis of GD. DID is defined in the DSM 5^1^ as a disruption of identity characterized by 2 or more distinct personality states.

People suffering from DID describe different alter personalities (AP), which have different mannerisms and moods, often different handwriting, food preferences, or preferences in clothes and also different gender identities, which may differ from the assigned gender at birth, as seen in GD.^7^

Despite the fact that clinicians often think that DID is a rare syndrome, a prevalence rate of DID of 1%–3% of the population has been described.^7^ Accurate clinical diagnosis affords early and appropriate treatment. Self-report instruments such as the Dissociative Experiences Scale (DES),^8^ serve as potential screening measures for patients with DID. Clinician-administered instruments such as the Dissociative Disorders Interview Schedule (DDIS)^7^ and the Structured Clinical Interview for DSM-IV Dissociative Disorders-Revised (SCID-D-R)^9^ guide the clinician in making a firm diagnosis of DID.^10^

We will next describe a case report of a person suffering from DID and asking for treatment for GD. We will then provide a review of the literature on GD and DID, and give some advice concerning the MHA and care of patients suffering from DID and asking for GD treatment.

In the present article, we have decided to use a patient's assigned gender at birth when discussing case reports about patients with DID and without GD.

## Methods

### Case Report

The assessment and follow-up of a patient suffering for DID but asking for treatment for GD was described. The patient provided written informed consent.

### Literature Review

This review was carried out according to the guidelines of the Preferred Reporting items for systematic reviews and meta-analyses (PRISMA).^11^ A literature search was performed up to the December 23, 2020 using the combination of key words shown in Table 1, in the following online databases: PubMed, PsycInfo, and Embase databases.

**Table 1:** Keywords

Keywords	(dissociative OR “multiple personality” OR hysteri*) AND (“gender dysphoria” OR transsexual* OR trans-sexual* OR transgender OR “gender identity” OR “gender incongruence” OR transvesti*)

Duplicates were eliminated.

Two independent reviewers screened the abstracts, selected them using inclusion criteria, and analyzed the findings.

Studies were included if they reported data on gender nonconforming identities and dissociative disorders together.

We included all original studies regardless of their methodology, size of the research group, and the presence of the control group.

Studies were excluded if there were no data on gender both nonconforming identities and DID, and if they were reviews, thesis and letters.

Owing to the considerable heterogeneity of the studies and outcome measures, it was not possible to provide Forest plots. We used a narrative approach to synthesize the data from the studies included in this review.

## Results

### Case Report

A 27-year-old person, AMAB, was referred by his psychiatrist to our sexology unit for an evaluation of his GD. The patient questioned his gender and wondered whether he should begin a transition including a feminisation of the body. The patient was already in care by his general practitioner for a somatoform disorder.

During the first appointment of the MHA, the patient described a gender incongruence in relation to a stable female gender identity that he had felt since his adolescence. This made him feel the need, at times, to live in a female social role and in a female body. For this reason, he had been growing his hair for many years, and sometimes wore female clothes and behaved in a way to socially express his female gender in general attitudes and sexual behaviours. When his female gender identity was preponderant he would think about taking feminizing HT in order to have a body more in adequacy with his female gender. This wasn't a preoccupation when his female gender identity was not preponderant.

The patient's explanations in the first appointment sounded like GD in a person with gender nonconforming identity. During the other appointments of the MHA, however, after asking for more precision, the patient described the different personalities he had within himself. He described 8 distinct personalities with different gender identities (2 female and 6 male). Each of these personalities had different characters. The patient also described numerous memory lapses sometimes linked to traumatic events, and was experiencing difficulty to fit in socially and professionally.

The patient had been adopted during childhood and had a leukemia as a child with long hospitalizations and treatments. Five years before the first appointment in our sexology unit, he had begun to suffer from a somatoform disorder, with tremors, pains and even paralysis of the legs for 6 months.

Only the female personalities adhered to his request for HT for GD.

After collecting all this information, the diagnosis of DID was first suspected and then confirmed through a clinical interview using DSM 5 criteria, the DES^8^ and the SCID-D-R.^9^

Although the patient described some criteria for GD (ie, the strong desire for the secondary sex characteristics of the other gender, the desire to be of the other gender different from the assigned gender, the desire to be treated as the other gender or an alternate gender different from the assigned gender and the conviction to have typical feelings and reactions of the gender different from the assigned gender), it appears here that the DID diagnosis takes precedence. The patient accepted extra sessions to discuss the possibilities of HT for his gender incongruence, but, as the sessions progressed, the different personalities understood that the wish for HT was actually a wish coming from his DID which needed psychotherapy. The patient was able to let go of his wish for HT. The patient is currently in psychotherapy for his DID.

### Literature Review

**Figure 1:**
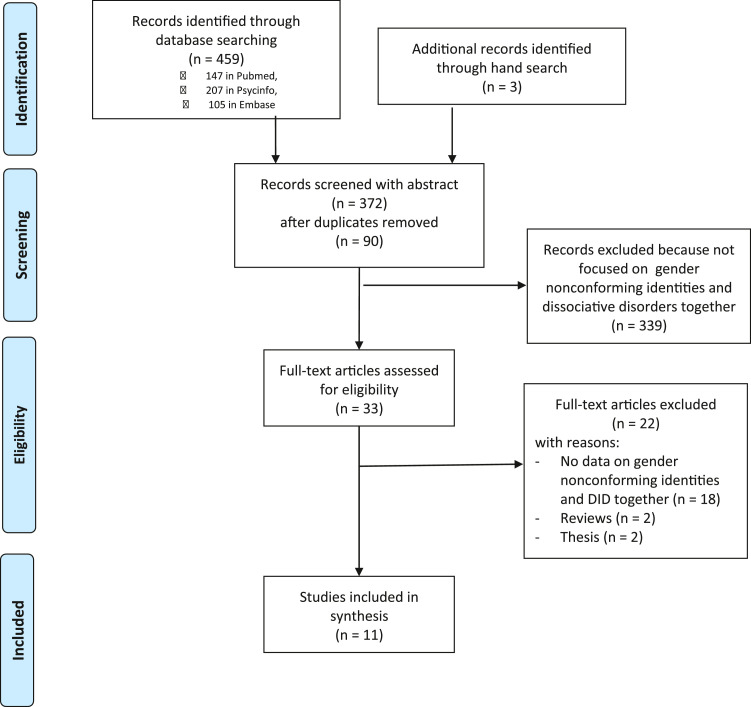
Study retrieval process.

Figure 1 details the study retrieval process.

We found 459 results (147 in Pubmed, 207 in Psycinfo, 105 in Embase) using a systematic search in the databases.

90 duplicates (38 in Psycinfo database and 52 in Embase database) were eliminated.
2 articles were added to results through hand search.

After selection using inclusion criteria, 32 abstracts were considered for full-text screen.

After selection using exclusion criteria, 11 articles were included in the systematic review as shown in Table 2.

**Table 2:** Literature review results

Authors	Study type	Sample size	Outcome measures	Main considerations
Kersting et al. 2003^13^	Between design (3 clinical group vs CG)	N = 475 - 41 GD - 29 AMAB - 12 AFAB - 115 psychiatric inpatients - 59 dissociative disorders - 260 CG	For GD: - DSM-IV For DID: - DES - SCID-D-R	In GD group: - 0% DID In psychiatric inpatients group: - 0,87 % DID In dissociative disorders group: - Not mentioned In CG: - Not mentioned
Colizzi et al. 2015^14^	Within design (only clinical group)	N = 118 GD - 82 AMAB - 36 AFAB	For GD: - DSM-IV TR For DID: - DDIS - DES	0.8% DID (1 AMAB)
McDuffie & Brown, 2010^15^	Within design (only clinical group)	N = 65 GD veterans - 91% AMAB - 9% AFAB	For GD: - Semi-structured clinical interview - For DID: - Semi-structured clinical interview	1.5% DID
Mun et al. 2020^19^	Case report	1 AFAB 20 year-old	For GD: - Clinical assessment For DID: - Clinical assessment	Diagnosis: GD and DID - Diagnosis of DID before the assessment for GD - DID with 6 personalities, most of them male - By age of 18, diagnosis of DG and initiation of HT - By age of 20, reduction in distress of his physical body and GD symptoms but increase in hostility and violence, as well as predominance of 2 existing male AP - Sexual abused in childhood
Schwartz & Pearl, 1988^21^	Case report	1 AMAB 33 year-old	For GD: - Clinical assessment For DID: - Clinical assessment	Diagnosis: GD and DID - By age of 25, diagnosis of GD and GAS - Many years after GAS, diagnosis of DID - DID with different males and females AP - GAS requested by females AP, also if male personalities were against GAS - After GAS, some males AP hated the feminized body and felt raped when having sex intercourse with men - As the psychotherapy for DID has progressed, males AP accepted GAS - Sexual abused in childhood
Saks, 1998^20^	Case report	1 AFAB 55 year old	For GD: - Clinical assessment For DID: - Clinical assessment	- Diagnosis: GD and DID - By age of 32 to 34, diagnosis of GD, withholding information about AP, and 3 GAS - 10 years after GAS, diagnosis of DID - DID with 33 personalities, some males and some females, with male personalities dominating, who wished GAS and kept the others away in order to have GAS - Persistence of males and females AP after GAS - Sexual abused in childhood
Money & Primrose, 1968^18^	Case report	1 AMAB no age specified	For GD: - no formal diagnosis, only clinical description with all symptoms For DID: - no formal diagnosis, only clinical description with all symptoms	- Diagnosis: GD and DID - Age when GD and DID diagnosis not specified - GAS - DID with 1 female AP who didn't knowledge to have had a GAS
Money, 1974^17^	Case report	1 AMAB no age specified	For GD: - no formal diagnosis, only clinical description with all symptoms For DID: - no formal diagnosis, only clinical description with all symptoms	- Diagnosis: GD and DID - Age when GD and DID diagnosis not specified - DID with 1 female AP - Prepared for GAS, but no information if GAS was performed
Becker et al. 1999^22^	Case report	1 AMAB 28 year-old	For GD: - Clinical assessment For DID: - SCID-D	- Diagnosis: Before GD, then corrected for DID - Age when GD and DID diagnosis not specified - After diagnosis of GD, began a follow-up for GD - 6 months after the beginning of the follow-up, real life experience in the female social role and wish for a GAS - 1 year after the beginning of the follow-up, the patient stabbing of a domina-prostitute
				- After crime, the diagnosis was corrected for DID - DID with 1 female AP
Modestin & Ebner, 1995^23^	Case report	Case report	For GD: - DSM-III-R criteria For DID: - DES - DDIS	- Diagnosis: Before GD, then corrected for DID - By age of 20, first diagnosis of GD, then of DID - After starting follow-up for GD, hospitalization for depression, and the diagnosis was corrected for DID - DID with 3 AP (2 female and 1 male). Male AP predominated and asked for a GAS, which was in contrast with females AP, what made feel the patient desperate and suicidal - During the psychotherapy proposed after DID diagnostic, the patient came to realize that the body dysphoria was in relation with DID and dropped the idea of GAS
Steinberg, 1995^24^		1 AMAB No age specified	For GD: - Clinical assessment For DID: - SCID-D	- Diagnosis: DID - DID with a female AP - Even if female AP asking for GAS, no diagnosis of GD
Soldati et al.^(in submission)^		1 AMAB 27 year-old	For GD: - Clinical assessment For DID: - DSM 5 criteria DES - SCID-D-R	- Diagnosis: DID - DID with 6 males personalities and 2 female AP - Even if 2 female AP asking for HT, no diagnosis of GD - During the psychotherapy proposed after DID diagnostic, the patient came to realize that the body dysphoria was in relation with DID and dropped the idea of HT

AFAB = Assigned Female At Birth; AMAB = Assigned Male At Birth; AP = Alter personality; CG = Control group; DDIS = Dissociative Disorders Interview Schedule; DES = Dissociative Experience Scale; DID = Dissociative Identity Disorder; GAS = Gender Affirming Surgeries; GD = Gender dysphoria; HT = Hormone Therapy; SCID-D = Structured Clinical Interview for DSM-IV Dissociative Disorders; SCID-D-R = Structured Clinical Interview for DSM-IV Dissociative Disorders–Revised

Three articles were within studies in GD population showing a prevalence of DID of respectively 0%^12^, 0.8% ^13^ and 1,5%.^14^

Five articles^15^, ^16^, ^17^, ^18^, ^19^ were case reports of patients with both diagnoses of GD and DID.

Mun's case report^17^ was an AFAB who was diagnosed with DID before the assessment for GD. By the age of 18, GD was diagnosed too, and the patient initiated HT with testosterone. Two years after the beginning of HT, the patient reported a reduction in distress of physical body and GD symptoms but reported increased hostility and violence, as well as the increased predominance of 2 existing male AP.

In Schwartz's^19^ and Saks's^18^ case reports, the diagnosis of GD was made and the GAS were performed many years before finding out that the patient also suffered from DID. In Schwartz's case report,^19^ some of the male personalities hated the feminized body and felt raped when a female AP had sex intercourse with a man. As the psychotherapy for DID progressed, the male personalities accepted the transition. In Sak's case report^18^ it is reported that even after the GAS the male and female personalities continued to coexist.

Two older articles^15^^,^^16^ were both case reports of AMAB patients showing features of both GD and DID, but these articles don't mention a formal diagnostic. Both patients had a male gender identity with an AP with female gender identity. In the first case report,^16^ the patient underwent GAS. The female AP was gradually taking over, and the male personality believed that she would soon displace him completely.

Two further articles^20^^,^^21^ were case reports, where the GD diagnosis was first established, and counseling for GD was proposed. Subsequently, the diagnosis was changed for DID.

Becker's case report^20^ was about an AMAB patient, who reported a female gender identity from childhood, and was clinically diagnosed with GD and who began counseling for GD. Six months after the diagnosis, the patient began the real life experience in the female social role and expressed the wish for GAS. One year after the beginning of counseling, the patient suddenly and severely stabbed a domina-prostitute. Eventually the diagnosis was changed to DID, using validated instruments as the SCID-D.^22^ This patient had a male personality and another female AP who wanted GAS. The authors speculated that, counseling which supported the wish for GAS, had created a tension between the 2 personalities. The male personality part must have felt acutely threatened by the possibility of GAS and reenacted this threat in a real sadomasochistic encounter with the domina-prostitute. Furthermore, for the authors, this might also be a reenactment of non-conscious memories of real experienced childhood traumas.

Modestin's case report^21^ was about an AFAB patient, first diagnosed with GD. After starting counseling for GD, the patient was hospitalized for depression, and the diagnosis was changed to DID using the DES^8^ and the DDIS clinical interview.^7^ The patient described 4 personalities. The male AP had never been satisfied with his female body and asked for GAS, which was in contrast with the female personalities’ wishes, and this made feel the patient desperate and suicidal. During the psychotherapeutic treatment initiated after the DID diagnostic, the patient came to realize that the body dysphoria was in relation with the DID and dropped the idea of GAS.

In Steinberg's book,^22^ like in our case report, there is a description of 2 AMAB patients asking for HT, and GAS.^22^ After the initial assessment, a DID diagnostic was made and there were not enough criteria for a GD diagnosis. The patients had 1 or more male personalities and 1 or more female AP who declared having gender incongruence with their body and a desire to have GAS. As HT or GAS were not proposed, the patients underwent a psychotherapy for DID, and they concluded the HT and GAS were not suitable treatments for their disorders.

## Discussion

First and foremost, our review shows that the literature in this field is very limited.

Regarding the prevalence of DID in GD samples, 3 studies show a prevalence of 0%,^12^ 0.8%^13^ and 1,5%.^14^ It is generally estimated that the prevalence of DID is around 1%–3% in the general population^10^ which could mean that DID in GD sample is not higher than in the general population.

In the 5 case reports which are concerned with the comorbidity between GD and DID,^15^, ^16^, ^17^, ^18^, ^19^ only Mun et al^17^ describes a decrease in GD with HT, while Schwartz et al^19^ as well as Saks^18^ describe that tension remains between the personalities of different genders regarding GAS, including, in Schwartz's case report, the feeling of being violated in sexual intercourse after GAS.

In addition, the difficulty in making the differential diagnosis between GD and DID is evident in 4 case reports. In 2 of them, mistakes were made in establishing the diagnosis^20^^,^^21^ as GD was diagnosed instead of DID. Becker et al^20^ even suggested that a diagnosed GD instead of DID might be considered a serious medical mistake with considerable psychological side effects and leading to a crime because of the GD treatment sessions.

Steinberg's case report^22^ and the case report in this article describe AMAB patients who request for care for GD. However after a thorough MHA, the diagnosis of GD was not retained and the patients were able to benefit from psychotherapy for DID.

Regarding the difficulties in making the differential diagnosis between GD and DID, some authors have stressed that people with DID may frequently experience bewilderment or confusion in their gender identity, sexual orientation or more specifically sex characteristic of their body, because their different personalities have different genders. Particular importance should be attributed to this disorder in considering the differential diagnosis for GD.^13^^,^^22^

Some authors suggest that patients with DID describe a global identity disturbance, unlike patients with GD who focus more on the gender incongruence.^23^ Further difficulty in the assessment of DID in patients asking for GD treatments, is that GD is usually a self-diagnosis and that DID is frequently a delayed diagnosis. People with GD are typically open about their distress and ask for specific interventions. In contrast, people with DID frequently hide their symptoms and therefore these symptoms must actively be looked for.^21^ Sometimes AP who wish for surgery may actively try to keep other personalities away in order to obtain HT or GAS.^18^

Furthermore, as stressed by the International Society for the Study of Trauma and Dissociation (ISSTD),^24^ it is difficult to diagnose DID. This is primarily because of a lack of education among clinicians about dissociation, dissociative disorders, and the effects of psychological trauma. This leads to limited clinical suspicion about dissociative disorders and misconceptions about their clinical presentation. Often, instead of showing visibly distinct AP, a typical DID patient presents a mix of dissociative symptoms and other psychiatric symptoms, as posttraumatic stress disorder, depression, panic attacks, substance abuse, eating-disordered symptoms. The prominence of the latter, highly familiar, symptoms often leads clinicians to only diagnose these comorbid conditions. When this happens, the undiagnosed DID patient may undergo a long and frequently unsuccessful treatment for these other conditions.

Finally, almost all practitioners use standard diagnostic interviews and mental status examinations that do not include questions about dissociation or take a history of psychological trauma. Because DID patients rarely give information about their dissociative symptoms, the absence of a focused inquiry about dissociation prevents the clinician from diagnosing the disorder.

Concerning the appropriateness of offering HT or GAS to patients with both diagnoses of GD and DID, some authors stress that it is highly questionable that these patients should receive GAS until the DID has been treated, and stress the importance, in the sessions prior to GAS, of providing psychotherapy for the dissociative symptoms.^19^ Other authors propose that, while not requiring integration of the AP before GAS, at the very least there should be informed consent or a consensus of all the known AP.^18^

This review of the literature leads the authors to believe that the comorbidity of GD and DID cannot be considered an absolute contraindication for HT or GAS, since Mum's case report showed a benefit from HT and some case reports^16^^,^^18^^,^^19^ describe patients who seem to have found an equilibrium after GAS, but these cases pose ethical problems in terms of informed consent. A psychological follow-up seems indispensable to understanding the experiences of the different AP before deciding on a treatment for GD.

Regarding HT, Mun's case report^17^ points to a correlation between testosterone therapy and the expression of DID in an AFAB patient. Although the patient reported a reduction of GD after initiation of the HT, he reported an increase in hostility and agressivity, as well as an increased predominance of an existing male AP. The authors, after mentioning the known association between testosterone and the expression of aggression, point out the lack of knowledge about the effects of testosterone on AFAB patient suffering from comorbid DID, and speculate that testosterone therapy may adversely affect the experience of childhood trauma, often present in DID patients.

## Conclusion

This article provides the first systematic review on GD and DID and shows the importance of alerting mental health professionals to the risk of confusing DID with GD and the possibility that these conditions may coexist.

In addition, our review shows the complexity of following patients with a comorbidity of GD and DID, as well as the complexity of making the differential diagnosis between GD and DID.

Mental health professionals working with GD patients should include DID in their differential diagnosis, and, if there is any evidence of memory lapses or dissociation, the use of diagnostic tests such as the DES,^8^ DDIS,^7^ the SCID-D-R^9^ may be helpful, as recommended by the ISSTD.^24^

In patients seeking care for GD who have both GD and DID, it is important to offer a psychological follow-up in order to understand the experiences of the different AP. In this way, the patient may engage in a dialogue with their different AP in order to find the compromise that best suits the different AP before deciding on a treatment for GD.

It is important to stress that misdiagnosis of DID for GD and counseling for GD in a patient suffering from DID may have had psychological iatrogenic effects, as shown in Becker's case report.^20^ This would be in line with the complications highlighted in the literature about the psychotherapy of cases of unrecognized dissociative disorders, such as suicidal episodes, self-harm, psychotic deflections and reenactments of real trauma experienced in the therapeutic setting as well as the non-therapeutic space.^25^

If the diagnosis of DID is confirmed, the therapy of choice is psychotherapy according to the “Guidelines for treating dissociative identity disorder in adult” of the ISSTD.^24^ The treatment aims at establishing a trusting therapeutic relationship in which the patient can gain access to the different split-off personalities. The next step, is the reconstruction and integration of the traumatic experiences that led to the splitting of the personalities. Only after the integration of the different AP has taken place, can a reintegration into normal relations be possible.

There are several limitations to the possible interpretations stemming from the present literature review. The limitations are due to the small number of articles and the fact that the majority are case reports. In addition, the assessment is often only clinical, without validated assessment instruments, which creates an important potential for bias.

More studies are warranted to better understand the prevalence of coexistence of GD and DID, and the impact of GD counseling, HT and GAS on coexisting DID and, finally, the iatrogenic consequences of treating a patient mistakenly diagnosed with GD instead of DID.

The authors of this article refer to the version 7 of the standards of care (SOC) of the WPATH^3^ where a MHA is recommended to exclude that the GI is not accounted for by a mental disorder. There are reasons to believe that this recommendation will be revised in SOC-8. This would encourage clinicians to find respectful ways to investigate DID without a general screening of all patients. It will probably be beneficial to require comprehensive experience with gender identity issues by those who do the MHA, in order to identify when there is a need for a more extensive psychiatric examination.

## Statement of authorship

Conceptualization: Lorenzo Soldati; Methodology: Lorenzo Soldati; Data curation: Lorenzo Soldati, Roland Hasler, Nathalie Recordon, Marie Clément; Formal analysis: Lorenzo Soldati, Roland Hasler, Nathalie Recordon, Marie Clément; Writing – Original Draft: Lorenzo Soldati; Writing – Review & Editing: Lorenzo Soldati, John Kohl, Nader Perroud.
